# Supplementary material for: Effects of Haematococcus pluvialis Addition on the Sensory Properties of Plant-Based Meat Analogues
Source: Foods. 2023 Sep 15;12(18):3435. doi: 10.3390/foods12183435 (PMC10528005; doi:10.3390/foods12183435)
Supplement: Supplementary file 1 [file foods-12-03435-s001.zip › Table S1.pdf]

Table S1. Evaluate criteria and assign points of sensory property.

Sensory property	Evaluate criteria	Assign points
color	Moderate redness and brightness, uniform and close to the traditional meat.	8-10
	slightly darker or lighter, uneven can be overlooked.	6-8
	significant dark, uneven slightly	4-6
	Dark and uneven so that different to the traditional meat	2-4
	Color too dark or light, obviously different to traditional meat	0-2
odor	Slight fishy smell of soybeans and pleasant grassy aroma.	8-10
	Slight Soybean smell, pronounced grassy aroma but no pungent.	6-8
	soybean smell, slight pleasant grassy flavor	4-6
	Obvious soybean smell and fishy aroma	2-4
	Strong fishy of bean, fishy aroma of algae	0-2
