# Supplementary figures and images for: Effects of Haematococcus pluvialis Addition on the Sensory Properties of Plant-Based Meat Analogues
Source: Foods. 2023 Sep 15;12(18):3435. doi: 10.3390/foods12183435 (PMC10528005; doi:10.3390/foods12183435)

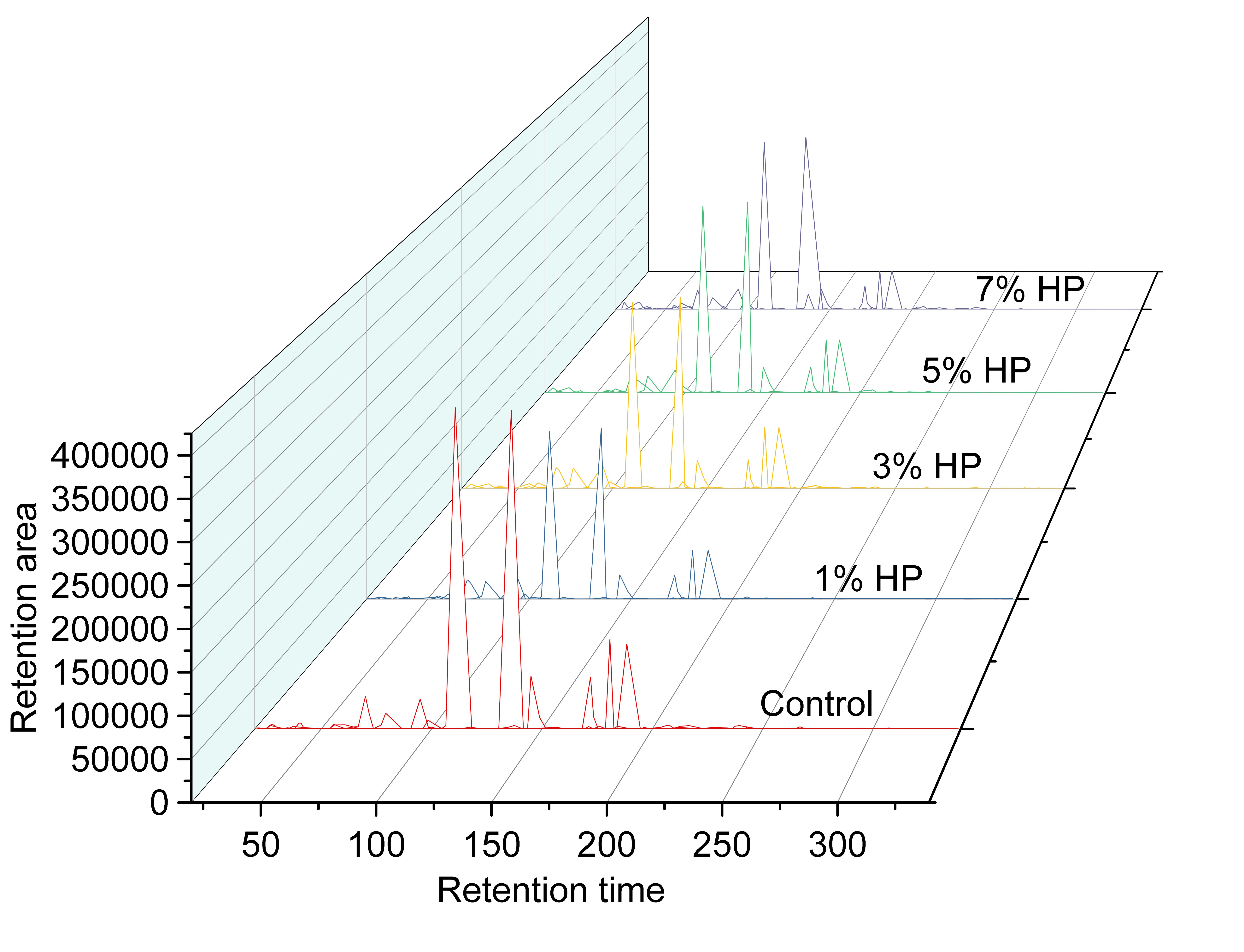

Supplement: Supplementary file 1 [file foods-12-03435-s001.zip › Figure S1.jpg]
